# Peak-Independent Cuffless Blood Pressure Monitoring Using a Smart Sock: The Role of Temporal Lag Modeling in Foot-Based PPG

**DOI:** 10.3390/s26041269

**Published:** 2026-02-15

**Authors:** Hamed Abdollahzadeh, Elisa Montaldi, Riccardo Olivieri, Paolo Esposito, Gianluca Barile, Giuseppe Ferri, Vincenzo Stornelli

**Affiliations:** Department of Industrial and Information Engineering and Economics, University of L’Aquila, 67100 L’Aquila, Italy; hamed.researcher.ml@gmail.com (H.A.); riccardo.olivieri1@graduate.univaq.it (R.O.); paolo.esposito2@graduate.univaq.it (P.E.); gianluca.barile@univaq.it (G.B.); giuseppe.ferri@univaq.it (G.F.); vincenzo.stornelli@univaq.it (V.S.)

**Keywords:** cuffless blood pressure monitoring, photoplethysmography (PPG), peak-independent features, temporal lag modeling, wearable sensors, smart textiles, machine learning regression, cardiovascular monitoring

## Abstract

Continuous blood pressure (BP) monitoring remains a major challenge in wearable healthcare systems, as conventional cuff-based sphygmomanometers are intermittent and unsuitable for long-term use. This study presents a Smart Sock platform for cuffless BP estimation using single-site photoplethysmography (PPG). Unlike approaches based on pulse transit time or fiducial point detection, the proposed framework relies on peak-independent features extracted from PPG and its first and second derivatives, capturing blood volume and hemodynamic dynamics in the lower limb. PPG signals from 60 participants were segmented into overlapping 30 s windows and processed through a unified preprocessing pipeline. A compact set of physiologically meaningful statistical and information-theoretic features was extracted from each window, and temporal lag modelling (5–15 s) was employed to encode short-term hemodynamic memory without explicit peak detection. Multiple regression models were assessed using leakage-safe cross-validation strategies. In a subject-independent diagnosis scenario, the system achieved errors of 8.60 mmHg for systolic BP and 6.42 mmHg for diastolic BP. In a monitoring scenario with single-point calibration, performance substantially improved, yielding mean absolute errors of 1.3–1.7 mmHg and R^2^ > 0.90. These results demonstrate that foot-based PPG, combined with peak-independent feature engineering and temporal context modeling, enables accurate and comfortable continuous personalized blood pressure monitoring after calibration, while subject-independent estimation remains more challenging.

## 1. Introduction

Hypertension is one of the leading risk factors for cardiovascular disease and premature mortality worldwide, affecting over 1.4 billion individuals [1]. Continuous and accurate blood pressure (BP) monitoring is therefore critical for the prevention and management of stroke, heart failure, and renal disease [2]. However, conventional cuff-based sphygmomanometers provide only intermittent measurements, and they are uncomfortable for prolonged use, limiting their suitability for ambulatory and long-term monitoring. These constraints have driven increasing interest in cuffless BP estimation approaches for wearable healthcare systems [3,4,5].

Photoplethysmography (PPG) is widely used in wearable devices due to its noninvasive nature, low cost, and ease of integration. In combination with electrocardiography (ECG) or dual-site PPG, PPG signals enable the estimation of pulse transit time (PTT) and pulse wave velocity (PWV), two indices closely associated with arterial stiffness and blood pressure through established biomechanical models, such as the Moens–Korteweg relationship [6,7,8,9]. Despite their physiological interpretability, these approaches typically require multiple sensors and accurate synchronization, thereby increasing system complexity and sensitivity to motion artifacts [7,10].

Single-site PPG methods have emerged as more practical alternative for wearable BP monitoring. Finger- and wrist-based devices have shown promising results [4,5], but frequently rely on fiducial point detection within the pulse waveform. In real-word conditions, fiducial features are often distorted by motion, variable contact pressure, and peripheral vasomotor changes, leading to reduce robustness and generalization performance. Recent wearable platforms have explored finger-based [11], ear-based [12], and ECG–PPG hybrid configurations [10]. Despite their potential, these solutions remain constrained by user compliance, motion sensitivity, and anatomical instability. In contrast, the lower limb offers a mechanically stable and physiologically informative sensing site. Foot arteries exhibit larger pressure amplitudes and more consistent waveform morphology, particularly during daily activities, making them well suited for long-term and unobtrusive vascular monitoring [13].

Despite growing interest in cuffless blood pressure monitoring, a fundamental challenge remains the development of wearable systems that are physiologically interpretable, robust to waveform distortion, and suitable for long-term deployment. Most existing single-site PPG approaches rely on explicit fiducial point detection, which limits robustness under real world conditions. To address these limitations, this study focuses on the development and validation of a cuffless blood pressure monitoring framework based on single-site, foot-mounted PPG, with the goal of improving robustness to waveform distortion through a peak-independent feature extraction pipeline applied to the PPG signal and its first and second derivatives. To account for the temporal nature of BP regulation, a temporal lag modeling approach is introduced to encode short-term hemodynamic memory across consecutive windows. Therefore, this design enables learning algorithms to track baseline shifts and vascular tone variation without explicit peak detection.

The main contributions of this study are as follows:

1. The design of a foot-based Smart Sock framework for BP monitoring using single-site PPG and derivative signals;
2. The introduction of a peak-independent feature engineering pipeline combining statistical, energy-based, and information-theoretic descriptors;
3. The development of a temporal lag modeling strategy incorporating multiple feature delays (5–15 s) to capture short-term hemodynamic memory and vascular dynamics;
4. The definition of a clinically motivated evaluation protocol distinguishing subject-independent diagnosis from subject-calibrated monitoring scenarios;
5. The proposal of a comparative analysis against state-of-the-art wearable BP systems, highlighting advantages in anatomical stability, motion robustness, and physiological interpretability.

Overall, this study positions foot-based single-site PPG as a viable and physiologically grounded alternative for cuffless blood pressure monitoring. By combining peak-independent feature extraction with temporal lag modeling, the proposed framework aims to improve robustness against waveform distortion while preserving interpretability and wearability. The following sections describe the system design, data acquisition protocol, feature engineering methodology, and experimental validation in both subject-independent and calibrated monitoring scenarios.

## 2. Materials and Methods

This section initially describes the hardware employed in this work. In particular, the chosen methods for signal acquisition and the circuit components are discussed. Furthermore, this section reports the machine learning pipeline and the methods chosen to perform linear regression on the acquired data for blood pressure estimation.

### 2.1. System Overview

**Figure 1:**
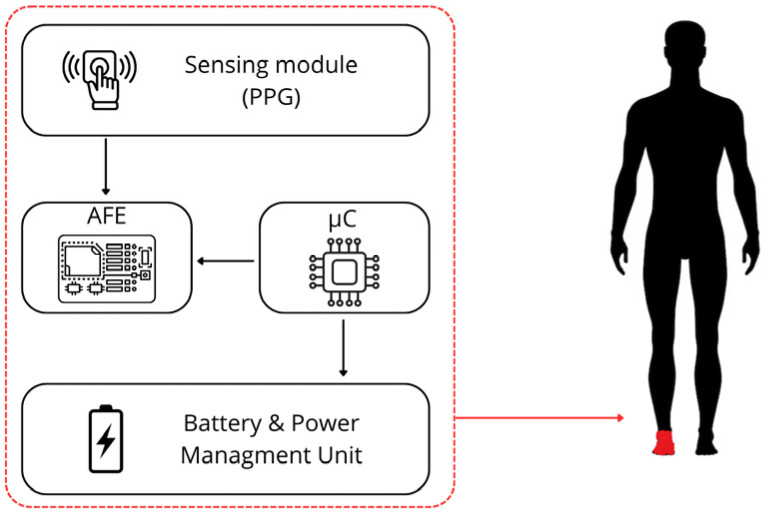
Schematic diagram of monitoring system.

This section describes the architecture of the proposed monitoring system, designed for the acquisition and processing of physiological signals. The overall configuration, shown in Figure 1, is composed of four main functional blocks: the sensing module (PPG), the Analog Front-End (AFE), the Microcontroller Unit (µC) and the battery and Power Management Unit (PMU). These subsystems are integrated within a compact, body-worn platform optimized for low-power operating and real-time data processing.

The sensing module consists of a textile-based smart sock integrating a PPG sensor (OpenBCI PPG, OpenBCI, Brooklyn, NY, USA) positioned on the foot. The sock must be fabricated to ensure comfortable wearability and stable skin contact during daily activities, enabling continuous optical monitoring of blood volume changes in the lower limb.

Photoplethysmography is an optical, non-invasive technique used to measure variations in blood volume within biological tissues, particularly in the microvascular bed [14,15]. Its operating principle relies on illuminating the skin using light-emitting diodes (LEDs), typically operating in the infrared, red or green wavelength ranges, and measuring the reflected or transmitted light through a photodiode [16]. Since blood exhibits different optical absorption properties compared to surrounding tissues, each cardiac cycle generates a characteristic pulsatile waveform in the recorded signal [14,16].

PPG technology is widely employed in several biomedical applications, including continuous cuffless blood pressure monitoring [14,16], oxygen saturation (SpO_2_) estimation [15], and heart rate and respiratory rate assessment [14]. In recent years, PPG sensors have been broadly integrated into wearable devices such as smartwatches, wristbands and fitness trackers [14,15], including implementations based on the ESP32-WROOM module [17,18,19,20,21,22,23]. Moreover, advanced developments include sensor arrays specifically designed for localized vascular assessments, for instance when applied to the plantar surface of the foot, enabling region-specific hemodynamic monitoring [15].

In this work, the PPG signal is acquired in a single-site configuration and processed without relying on pulse timing, fiducial point detection, or PTT estimation, to enable peak-independent BP inference. The embedded PPG sensing module operates in reflectance mode using near-infrared illumination, selected for its deeper tissue penetration and enhanced signal stability in plantar vascular measurements.

The analog signals generated by the PPG sensor are first processed by the AFE, which performs signal conditioning operations such as amplification, filtering and impedance matching. This stage enhances the signal-to-noise ratio and prepares the signal for accurate analog-to-digital conversion.

Conditioned signals are subsequently sampled by the microcontroller unit, which manages data acquisition, buffering, and wireless streaming to an external workstation for offline processing. All feature extraction and machine learning operations are performed off-device during post-processing. The µC also controls communication with external devices and coordinates the operation of the sensing and power subsystems.

Finally, the battery and the PMU handle power conversion, voltage regulation and battery charging, ensuring a stable and efficient energy supply to all electronic components. The PMU is designed to minimize power consumption and support long-term wearable operation.

### 2.2. Hardware Design and Implementation

The hardware implementation of the proposed system is based on the development and realization of a fully custom-designed printed circuit board (PCB), which integrates all the key functional units required for signal acquisition, processing and power management. The board hosts the µC, based on the ESP32-WROOM-32 module (Espressif Systems, Shanghai, China), which provides high-performance processing, integrated Wi-Fi connectivity and multiple GPIO lines for peripheral interfacing.

The PCB also incorporates a USB-to-UART communication interface to enable programming and data exchange between the ESP32 microcontroller and an external computer. The interface includes level-shifting and power management circuitry, ensuring compatibility with both USB (5 V) and ESP32 logic levels (3.3 V). Protection components, such as transient suppressor diodes (SP0503) and decoupling capacitors, are integrated to prevent overvoltage and noise injection from the USB line. This interface enables for both firmware upload and real-time data acquisition at a baud rate of 256,000 bps, making the board fully autonomous and easy to interface during development and testing.

The power management architecture has been specifically designed to ensure stable operation and minimize noise propagation within the analog measurement chain. The board is powered at 5 V supply line, which feeds a set of low-dropout (LDO) voltage regulators generating two independent 3.3 V rails: one dedicated to the digital domain (microcontroller and communication interfaces) and the other to the analog front-end circuitry. To further reduce noise coupling, the analog and digital grounds have been isolated and decoupled and they are connected only in DC but separated for AC components. This configuration effectively filters out high-frequency disturbances that could degrade measurement accuracy.

The PCB is modular and expandable, providing a 32-channel analog input array that can be configured for single-ended or differential acquisition (up to 16 differential inputs) with a 16-bit ADC resolution. This flexibility supports the connection of a wide range of sensors for connection to expansion boards, such as the IMU and an analog interface, so it facilitates system scalability and integration without additional wiring complexity.

**Figure 2:**
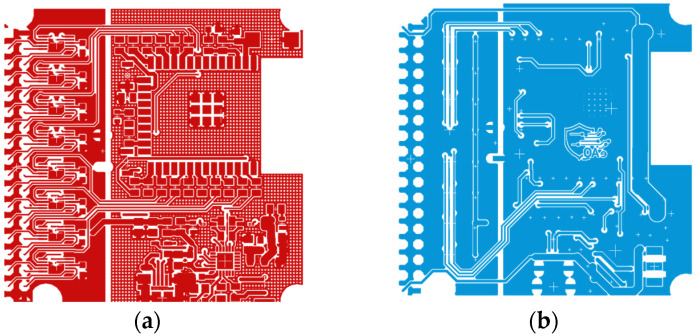
PCB layout designed in Fusion 360: (**a**) top layer (**b**) bottom layer.

**Figure 3:**
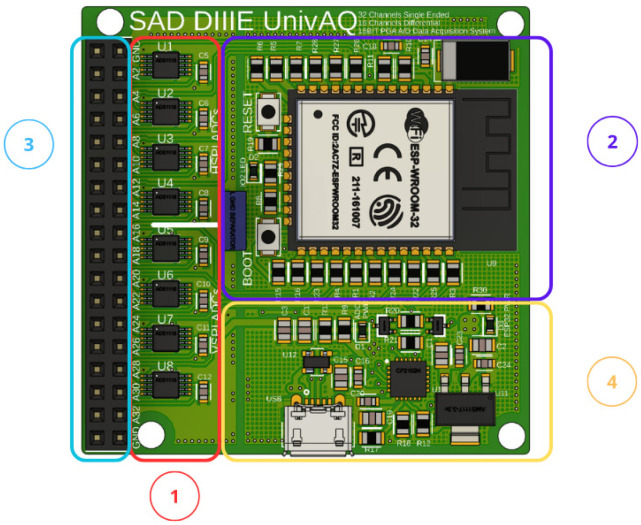
Annotated view of the top layer of the custom-designed PCB highlighting the main blocks: (1) Analog Input Channels for signal acquisition; (2) ESP32-WROOM-32 Module; (3) Expansion headers for peripheral connection; (4) USB-to-UART interface and voltage regulators.

The board is fully custom-designed, optimized for compactness, low power consumption and high signal integrity. Figure 2 shows the top and bottom layers of the PCB layout designed in Autodesk Fusion 360, while Figure 3 presents the manufactured prototype.

### 2.3. Participants and Data Collection

A total of 60 adult participants were initially recruited for this study. After excluding recordings with incomplete reference blood pressure measurements or insufficient signal quality, data from 56 participants were retained for subsequent analysis. All participants provided written informed consent prior to enrollment, and the study protocol complied with institutional ethical guidelines.

Physiological data were acquired under resting conditions to minimize confounding effects from motion-induced artifacts and acute cardiovascular stress; no measurements during motion or during physical exercise were included in this study. Each participant wore the proposed foot-based Smart Sock, which continuously recorded single-site PPG signals from the plantar region of the foot. The foot was selected as the sensing location due to its anatomical stability, pronounced pulse pressure, and suitability for long-term wearable monitoring.

PPG signals were sampled at 100 Hz, providing sufficient temporal resolution for waveform morphology analysis while maintaining computational efficiency for wearable-oriented signal processing. Each recording session lasted approximately 2–6 min, resulting in inter-subject variability in recording duration. This variability naturally produced differing numbers of analysis windows per participants and motivated the subject-aware modeling and aggregation strategies described later in this work.

Reference systolic blood pressure (SBP) and diastolic blood pressure (DBP) values were obtained for each participant using a clinically validated automated cuff-based sphygmomanometer immediately prior to PPG acquisition. Simultaneous cuff inflation during PPG recording was intentionally avoided, as cuff-induced arterial occlusion and transient hemodynamic perturbations are known to significantly alter distal perfusion and PPG waveform morphology, particularly at the foot. Over the short recording interval, blood pressure was assumed to remain physiologically stable; therefore, the cuff-based measurement was considered a valid subject-level reference for all PPG windows extracted from the corresponding recording. No continuous or beat-to-beat blood pressure measurements were assumed; instead, SBP and DBP were treated as subject-level physiological states associated with each participant during the acquisition session.

The resulting dataset consisted of continuous foot-based PPG recordings paired with subject-level SBP and DBP reference values. This data structure, characterized by multiple overlapping signal windows associated with a single BP measurement per subject, necessitated careful handling of temporal dependence and inter-subject variability in both feature engineering and model evaluation. These considerations directly guided the windowing strategy, temporal lag matrix construction, and leakage-safe cross-validation protocols adopted in this study.

### 2.4. Signal Processing

All acquired PPG signals were processed offline using a unified Python-based framework (Python 3.11) aimed to suppress noise, standardize signal morphology, and ensure physiologically meaningful input for downstream feature extraction and machine learning. The signal-processing pipeline was intentionally designed to be peak-independent, avoiding reliance on fiducial point detection, which is known to degrade under motion artifacts, distal sensing locations, and variable sensor–skin contact conditions.

#### 2.4.1. Preprocessing and Filtering

Raw PPG signals were first detrended to remove slow-varying baseline components arising from sensor drift and postural changes. Subsequently, the signals were bandpass filtered using a zero-phase Chebyshev Type II filter with cutoff frequencies of 0.4–8 Hz. This frequency range was selected to preserve the fundamental cardiac oscillations and relevant harmonic content while attenuating baseline wander and high-frequency noise.

The use of a zero-phase filtering strategy ensured that no phase distortion was introduced, thereby preserving the temporal structure of the waveform for downstream derivative computation. Finally, each windowed PPG segment was standardized using z-score normalization:

(1) $$x_{norm}\left(t\right)=\frac{x\left(t\right)-\mu_{x}}{\sigma_{x}}$$

where $\mu_{x}$ and $\sigma_{x}$ denote the mean and standard deviation of the windowed signal, respectively. This normalization reduced inter-subject amplitude variability caused by differences in tissue composition, sensor contact pressure, and vascular depth, enabling robust comparison across participants.

#### 2.4.2. Derivative Signal Construction

To enhance sensitivity to vascular dynamics beyond raw blood volume changes, derivative representations of the PPG waveform were computed. The first derivative of the normalized PPG signal, referred to as the Velocity Plethysmogram (VPG), emphasizes the rate of change in blood volume and provides insight into velocity-related components of blood volume changes, while the second derivative, known as the Acceleration Plethysmogram (APG), emphasizes acceleration-related waveform components associated with arterial compliance and stiffness. To mitigate noise amplification caused by numerical differentiation, both VPG and APG signals were smoothed using a Savitzky–Golay filter (Python 3.11, Python Software Foundation, Wilmington, DE, USA; SciPy 1.11, SciPy Developers, Austin, TX, USA) with a polynomial order of 3 and a frame length of 11 samples at a 100 Hz sampling rate. This configuration preserves waveform morphology while reducing high-frequency artifacts. These derivative signals enabled the extraction of complementary physiological information without reliance on fiducial point detection or explicit pulse segmentation.

#### 2.4.3. Signal Quality Assessment

Rather than employing explicit heart rate tracking or cadence-based rejection, signal quality was evaluated using window-level statistical criteria derived from the PPG, VPG, and APG signals. Windows showing abnormal amplitude ranges, excessive variance, or unstable derivative behavior were excluded from further analysis.

This peak-independent quality control strategy ensured that only physiologically plausible segments were retained while avoiding the failure modes commonly associated with fiducial detection under motion or distal sensing conditions. By operating directly on signal statistics, the approach remains robust to waveform distortions typical observed in foot-based PPG measurements.

#### 2.4.4. Windowing Strategy

Each continuous PPG recording was segmented into overlapping windows of 30 s with a step size of 5 s. This window duration was selected to balance cardiovascular stationarity with temporal resolution, and it is consistent with prior work on PPG-based blood pressure estimation [4,5].

The overlapping windowing scheme increased the effective number of training samples while enabling the modeling of short-term hemodynamic trends across consecutive windows. Importantly, all windows derived from a given subject were associated with the same reference SBP and DBP values obtained from cuff-based measurements.

#### 2.4.5. Final Dataset Construction

Following preprocessing, derivative computation, windowing, and quality control, a total of 1110 valid PPG windows were retained across all subjects. Each window consisted of synchronized PPG, VPG, and APG signals and was labeled with the corresponding subject-level SBP and DBP values.

This window-level dataset formed the foundation for subsequent peak-independent feature extraction, temporal lag matrix construction, and machine learning-based blood pressure modeling.

### 2.5. Feature Extraction and Engineering

Feature extraction was designed to capture physiologically meaningful information from foot-based photoplethysmography signals while avoiding explicit reliance on fiducial point detection. Given the susceptibility of distal PPG waveforms to motion artifacts, signal attenuation, and inter-subject variability, a fully peak-independent strategy was adopted. All features were extracted from the normalized PPG signal and its first and second derivatives to jointly characterize blood volume dynamics, flow velocity, and pulse wave acceleration, and independently within each validated window, yielding a compact yet information-rich representation suitable for machine learning-based BP estimation.

#### 2.5.1. Statistical Morphological Features

Basic statistical descriptors were computed to summarize the distributional properties of the PPG, VPG, and APG signals within each window. These features quantify waveform symmetry, dispersion, and central tendency, which are known to reflect changes in vascular tone and arterial compliance.

For each signal modality, the following statistics were extracted:

- Mean;
- Standard deviation;
- Median;
- Interquartile range (IQR);
- Skewness.

The interquartile range was preferred over full-range measurements to reduce sensitivity to transient artifacts, while skewness provided insight into waveform asymmetry associated with systolic upstroke and diastolic decay.

#### 2.5.2. Energy-Based Features

To capture signal intensity and pulsatile strength independent of absolute amplitude, energy-based features were computed. For the PPG signal, the window-level energy was calculated as:

(2) $$E_{PPG}=\overset{N}{\underset{t=1}{\sum }}x^{2}\left(t\right)$$

where $x\left(t\right)$ denotes the normalized PPG signal and N is the number of samples per window.

In addition, a kinetic energy-inspired descriptor (KTE) was computed using the squared VPG signal to emphasize rapid blood volume changes associated with systolic ejection:

(3) $$KTE_{PPG}=\overset{N}{\underset{t=1}{\sum }}{\left(\frac{dx\left(t\right)}{dt}\right)}^{2}$$

For both energy measures, robust dispersion statistics (e.g., interquartile range) were retained rather than absolute values, improving robustness to sensor contact variability and inter-subject differences.

#### 2.5.3. Information-Theoretic Features

To quantify waveform complexity and irregularity, entropy-based features were extracted from the PPG and APG signals. Shannon entropy was computed from amplitude histograms within each window:

(4) $$H=-\underset{i}{\sum }p_{i}\log\left(p_{i}\right)$$

where $p_{i}$ represents the probability of the signal amplitude falling within the i-th bin.

Entropy features capture changes in pulse regularity and morphological variability that may arise from alterations in vascular resistance, autonomic regulation, or peripheral perfusion. These descriptors complement traditional statistical features by characterizing signal organization rather than magnitude.

#### 2.5.4. Zero-Crossing and Shape Descriptors

Zero-crossing rates (ZCRs) were computed for derivative signals (VPG and APG) to characterize waveform oscillatory behavior and curvature changes. The ZCR measures the number of sign changes within a window and serves as a proxy for pulse waveform sharpness and complexity.

Concerning distal sensing locations such as the foot, where waveform distortion is common, zero-crossing-based descriptors provide a robust alternative to peak timing features and are less sensitive to absolute amplitude or baseline drift.

#### 2.5.5. Temporal Lag Feature Matrix Construction

Blood pressure regulation exhibits temporal dependence due to vascular tone adaptation, autonomic feedback, and short-term hemodynamic memory. To explicitly model these dynamics, a temporal lag matrix was constructed by augmenting each window’s feature vector with delayed versions of selected features from preceding windows.

Specifically, for a given feature $f\left(t\right)$, lagged features were defined as:

(5) $$f_{lag_{k}}\left(t\right)=f\left(t-k\right),\;\;\;\;\;k\;\epsilon \;\left\{1,2,3\right\}$$

corresponding to delays of 5–15 s given the 5 s window step size.

This lag formulation allows learning algorithms to capture baseline shifts, slow vascular adaptations, and pulse stability trends without relying on explicit beat-to-beat timing or pulse transit time estimation. Windows lacking sufficient prior history were excluded to ensure temporal consistency.

#### 2.5.6. Final Feature Set

The final feature set comprised a hybrid combination of:

- Statistical morphology descriptors;
- Energy-based and kinetic energy features;
- Information-theoretic measures;
- Shape and zero-crossing features;
- Multi-scale temporal lag features.

This resulted in a compact yet expressive feature matrix that balances physiological interpretability with robustness to noise and motion artifacts. The engineered features served as inputs to all subsequent machine learning models described in Section 2.6.

### 2.6. Machine Learning and Physics-Compliant Modeling Framework

Blood pressure estimation from PPG constitutes a fundamentally ill-posed inverse problem, as multiple hemodynamic states may generate similar peripheral pulse waveforms. To address this challenge, we adopted a modeling framework that integrates data-driven machine learning with physiologically motivated feature engineering and evaluation protocols designed to reflect real-world clinical use cases.

Rather than relying on explicit pulse timing or pulse transit time computation, the proposed framework learns a nonlinear mapping between peak-independent features derived from foot-based PPG signals and reference SBP and DBP values.

#### 2.6.1. Feature–Target Mapping Formulation

Let $x_{i,t}\in {\mathbb{R}}^{d}$ denote the feature vector extracted from the t-th window of subject i, where d represents the total number of engineered features, including lagged components. Each window is associated with a subject-level reference blood pressure value:

(6) $$y_{i}^{SBP},\;y_{i}^{DBP}\;\epsilon \;\mathbb{R}$$

The learning objective is to estimate a regression function $f\left(\cdot \right)$ such that:

(7) $$\hat{y_{i,t}}=f\left(x_{i,t}\right)$$

where ${\hat{y}}_{i,t}$ denotes the predicted blood pressure for window t of subject i.

Because reference BP measurements are available only at the subject level, multiple windows share the same ground-truth label. This data structure motivates both leakage-safe cross-validation and subject-level aggregation strategies, described in subsequent sections.

#### 2.6.2. Temporal Lag Modeling and Hemodynamic Memory

Blood pressure regulation is governed by physiological feedback mechanisms operating over multiple time scales, including baroreflex control, smooth vascular muscle tone, and autonomic modulation. A single short PPG window therefore provides only a partial snapshot of the underlying hemodynamic state.

To incorporate in the model the short-term hemodynamic memory, a temporal lag matrix was constructed by augmenting each feature vector with delayed versions of selected features. For a base feature $f_{j}\left(t\right)$, the lagged representation is defined as:

(8) $$f_{j}^{lag}\left(t\right)=\left[f_{j}\left(t\right),f_{j}\left(t-\Delta \right),\;f_{j}\left(t-2\Delta \right),\;f_{j}\left(t-3\Delta \right)\right]$$

where Δ=5 s corresponds to the window step size. This formulation captures trends over a 5–15 s horizon, which aligns with known short-term cardiovascular regulation dynamics.

By using lagged feature representations, instead of raw signal histories, the model considers temporal context while remaining computationally efficiency and improved robustness to noise.

#### 2.6.3. Machine Learning Regressors

A diverse set of regression models was evaluated to capture both linear and nonlinear relationships between PPG-derived features and BP. All models were trained independently for SBP and DBP estimation.

Tree-based ensemble methods were selected due to their robustness in feature scaling, their ability to model nonlinear interactions, and their interpretability via feature importance analysis.

Random Forest regression was implemented as an ensemble of decision trees trained using bootstrap aggregation, where predictions are obtained by averaging the outputs of individual trees:

(9) $$\hat{y}=\frac{1}{M}\overset{M}{\underset{m=1}{\sum }}f_{m}\left(x\right)$$

where M denotes the number of trees.

Extra Trees regression was also evaluated. This method follows a similar ensemble structure but introduces additional randomness in split selection, that reduces variance and improves generalization in the presence of noisy features.

Gradient Boosting regression was implemented as an additive model trained sequentially to minimize a loss function L:

(10) $$f_{k}\left(x\right)=f_{k-1}\left(x\right)+\eta \cdot h_{k}\left(x\right)$$

where $h_{k}$ is a weak learner and η is the learning rate.

Gaussian Process Regression was implemented to model uncertainty-aware nonlinear relationships between features and blood pressure. A Gaussian process defines prior functions:

(11) $$f\left(x\right)\;\sim \;GP\left(0,\;k\left(x,x^{\prime }\right)\right)$$

where $k\left(\cdot ,\cdot \right)$ is a kernel function encoding assumptions about smoothness and scale.

Three kernels were evaluated:

- Radial Basis Function (RBF);
- Rational Quadratic (RQ);
- Matérn 5/2.

The Rational Quadratic kernel is defined as:

(12) $$k_{RQ}\left(x,x^{\prime }\right)=\sigma^{2}{\left(1+\frac{{\left|x-x\prime \right|}^{2}}{2\alpha l^{2}}\right)}^{-\alpha }$$

where $\sigma^{2}$ denotes the signal variance, l is the characteristic length scale, and α controls the relative weighting between large and small-scale variations. The Rational Quadratic kernel can be interpreted as a scale mixture of RBF kernels with different length scales and it proved particularly effective at capturing multi-scale physiological variability, consistent with the heterogeneous nature of vascular dynamics across subjects.

#### 2.6.4. Leakage-Safe Evaluation Protocols

A critical methodological consideration in PPG-based BP estimation is the prevention of data leakage caused by overlapping windows from the same subject appearing in both training and testing sets. To address this issue, two distinct evaluation protocols were adopted, each reflecting a clinically meaningful usage scenario.

To simulate first-time system usage without prior subject calibration, a subject-independent evaluation strategy was implemented using GroupKFold cross-validation. In this configuration, all windows belonging to a given subject were assigned exclusively to either the training or testing set, ensuring complete subject separation between folds. Formally, for each fold k=1,…,K:

(13) $$S_{k}^{train}\;\cap \;S_{k}^{test}=\emptyset$$

where $S_{k}$ denotes the set of subject identifiers in fold k. This protocol evaluates the model’s ability to generalize across unseen individuals and represents the most challenging and clinically conservative scenario.

To emulate longitudinal monitoring after an initial calibration measurement, a subject-calibrated evaluation protocol was also considered using a StratifiedKFold strategy at the window level. In this setting, the model is allowed to observe prior windows from the same subject during training, reflecting personalized tracking rather than cross-subject generalization. This scenario closely resembles realistic wearable use, where an initial cuff-based measurement may be available to anchor subsequent continuous estimates.

#### 2.6.5. Subject-Level Aggregation

Since arterial blood pressure does not exhibit substantial changes on a second-by-second basis under resting conditions, window-level predictions were aggregated to obtain a single representative estimate per subject. For subject i, the final predicted blood was computed as:

(14) $$\hat{y_{i}}=median_{t\in T_{i}}\left(\hat{y_{i,t}}\right)$$

where $T_{i}$ represents the set of all valid windows extracted from subject i, and $\hat{y_{i,t}}$ represents the model prediction associated with the t-th window.

Median aggregation was preferred over mean aggregation due to its increased robustness to outlier windows, which may arise from transient motion artifacts, momentary sensor displacement, or local signal distortions. This choke ensures that the final subject-level estimate reflects the dominant hemodynamic state while minimizing the influence of spurious predictions.

#### 2.6.6. Evaluation Metrics

Model performance was quantified using standard regression metrics computed at the subject level.

Specifically, the Mean Absolute Error (MAE) was used to measure the average magnitude of prediction errors:

(15) $$\mathrm{MAE}=\frac{1}{N}\overset{N}{\underset{i=1}{\sum }}\left|y_{i}-\hat{y_{i}}\right|$$

where *N* denotes the number of subjects, $y_{i}$ the reference blood pressure value, and $\hat{y_{i}}$ the aggregated model prediction.

The Root Mean Squared Error (RMSE) was also reported to penalize larger deviations more strongly:

(16) $$\mathrm{RMSE}=\sqrt{\frac{1}{N}\overset{N}{\underset{i=1}{\sum }}{\left(y_{i}-\hat{y_{i}}\right)}^{2}}$$

In addition, the coefficient of determination ($R^{2})$ was computed to assess the proportion of variance in the reference measurements explained by the model:

(17) $$R^{2}=1-\frac{\sum {\left(y_{i}-\hat{y_{i}}\right)}^{2}}{\sum {\left(y_{i}-\bar{y}\right)}^{2}}$$

where $\bar{y}$ represents the mean of the reference blood pressure values.

Unless otherwise specified, all metrics were reported at the subject level unless otherwise specified.

#### 2.6.7. Physiological Reliability Check

To ensure physiological plausibility, model outputs were systematically examined for consistency with known BP ranges and expected trends. Predictions exhibiting implausible excursions, excessive intra-subject variability, or abrupt fluctuations across consecutive windows were further analyzed to identify potential feature extraction or modeling artifacts.

This reliability assessment reinforces the suitability of the proposed framework for wearable deployment, where safety and interpretability are essential requirements.

## 3. Results

### 3.1. Signal Quality and Dataset Characteristics

This section summarizes the quality of the acquired signals and the main characteristics of the constructed dataset, providing insight into the reliability of the proposed sensing platform and the suitability of the data for BP modeling.

**Figure 4:**
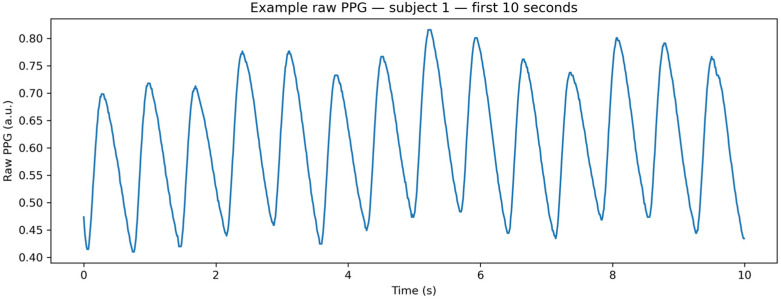
Representative 10 s segment of raw foot-based PPG acquired from the Smart Sock, illustrating preserved pulsatile structure prior to preprocessing.

Figure 4 shows a representative 10 s segment of raw foot-based PPG acquired using the Smart Sock platform. Despite the distal sensing location, the signal exhibits a clearly distinguishable pulsatile structure with visible cardiac cycles, confirming the feasibility of lower-limb PPG acquisition for blood pressure estimation even prior to signal conditioning.

After preprocessing, including detrending, bandpass filtering, and normalization, pulse morphology was consistently recovered for all the participants.

**Figure 5:**
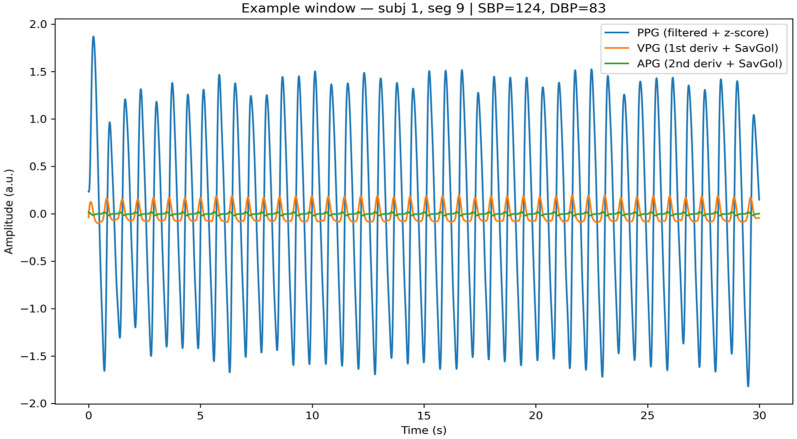
Example 30 s window after preprocessing showing the normalized PPG signal and its first and second derivatives, representing blood volume, velocity, and acceleration dynamics.

Figure 5 illustrates an example of 30 s analysis window together with VPG and APG. The derivative signals highlight blood flow velocity and acceleration dynamics, respectively, enabling physiologically meaningful characterization of vascular behavior without reliance on explicit pulse peak detection.

Across the entire dataset, a total of 1110 overlapping 30 s windows were extracted from PPG recordings collected from 56 subjects, using a fixed step size of 5 s. While overlapping windows improve temporal resolution and stabilize feature estimation, they also introduce strong correlation between adjacent samples. For this reason, two distinct evaluation protocols were adopted to ensure methodological rigor, each corresponding to a clinically relevant use case.

For subject-independent diagnosis, a GroupKFold strategy was employed, considering each subject as an independent group. All windows from a given subject were assigned exclusively to either the training or test set, ensuring that no overlapping segments from the same individual appeared in both sets. This protocol enforces subject-level separation and evaluates the model’s ability to generalize to previously unseen users, reflecting first-time use without prior calibration.

For longitudinal monitoring after calibration, a separate evaluation protocol was applied. In this scenario, all windows belonging to a subject were assumed to be available following a single reference cuff measurement, consistent with real-world continuous BP tracking. Under this assumption, stratified window-wise K-fold cross-validation was performed within the calibrated subject pool, preserving the SBP and DBP distribution across folds while assessing short-term tracking performance. Although adjacent windows overlap temporally, this evaluation quantifies the system’s ability to maintain consistent BP estimation throughout the entire 2–6 min recording duration, rather than cross-subject generalization.

To further improve robustness, model predictions were aggregated at the subject level prior to final evaluation, reducing the influence of individual noisy windows and transient artifacts. Together, this dual-protocol framework enables the use of overlapping windows for robust feature extraction while preserving strict methodological validity for both diagnostic and calibrated monitoring scenarios.

**Figure 6:**
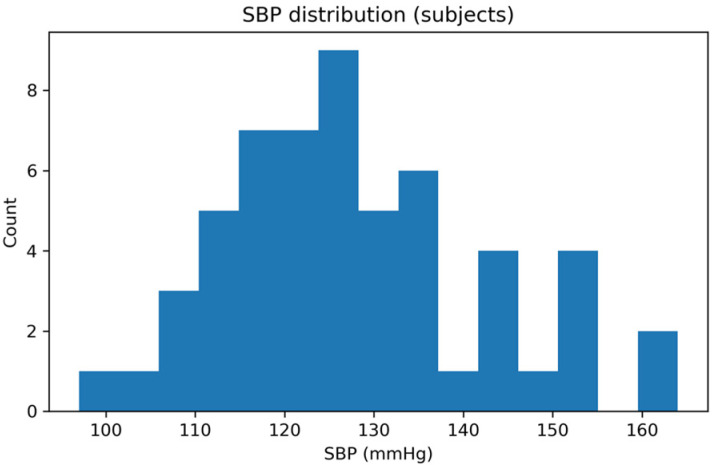
Distribution of reference SBP values across all subjects included in the final dataset.

**Figure 7:**
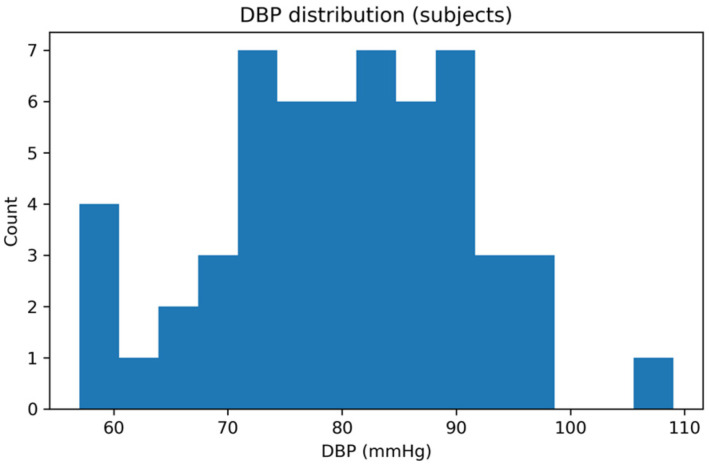
Distribution of reference DBP values across all subjects included in the final dataset.

Finally, the reference BP values spanned a wide physiological range. Figure 6 and Figure 7 report the distributions of SBP and DBP, respectively, confirming adequate representation of normotensive and hypertensive values within the dataset. This diversity supports a comprehensive evaluation of the proposed method across different cardiovascular conditions.

### 3.2. Feature Behavior and Temporal Dynamics

A compact set of peak-independent features was extracted from PPG, VPG, and APG signals, including statistical descriptors, energy-based measures, and information-theoretical metrics.

**Figure 8:**
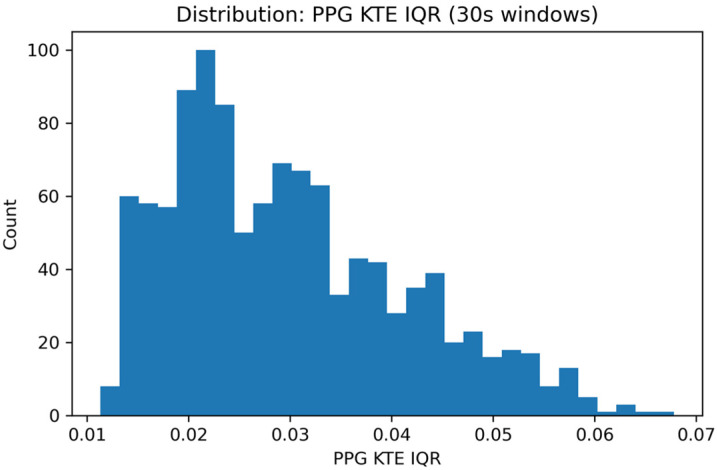
Distribution of the PPG Kaiser–Teager energy interquartile range (KTE-IQR) feature across all windows, illustrating its stability and suitability for temporal modeling.

Figure 8 shows the distribution of the interquartile range of the Kaiser–Teager energy (KTE-IQR) computed from the PPG signal, that emerged as one of the most informative features across multiple regression models.

To capture short-term hemodynamic memory, a temporal lag matrix was constructed by augmenting selected physiological features with delayed versions corresponding to 5, 10 and 15-s offsets. This approach enables each analysis window to incorporate recent vascular history, allowing the learning models to observe trends in baseline blood volume, pulse stability, and waveform morphology over time. By encoding temporal context directly into the feature space, the proposed framework models gradual changes in vascular tone and arterial stiffness without relying on explicit pulse timing or fiducial point detection.

### 3.3. Window-Level Blood Pressure Estimation

Window-level BP estimation was evaluated using leakage-safe, subject-wise cross-validation to assess subject-independent generalization. At the window level, predicted SBP and DBP values followed the overall physiological trends of the reference measurements; however, substantial variability was observed across individual windows. This dispersion reflects the inherent short-term fluctuations in peripheral hemodynamics and residual motion effects such as artifacts and local changes in sensor–skin coupling within 30 s segments. These results highlight the limitations of relying on single-window predictions for clinical interpretation and motivate the use of temporal context modeling and subject-level aggregation to improve robustness.

### 3.4. Subject-Level Aggregation and Diagnosis Scenario

**Figure 9:**
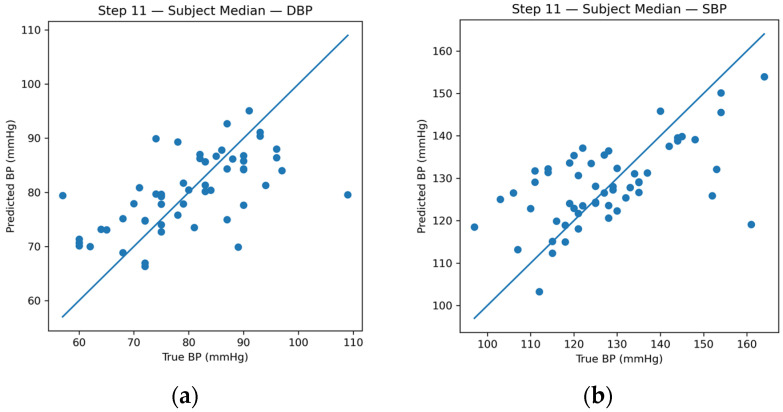
(**a**) Subject-level SBP predictions after median aggregation of window-level estimates. (**b**) Subject-level DBP predictions after median aggregation of window-level estimates.

In accordance with clinical BP assessment practices, window-level predictions were aggregated at the subject level using the median operator. Figure 9 shows subject-level predictions following aggregation, showing substantially reduced variability compared to the corresponding window-level estimates.

Under the subject-independent evaluation (diagnosis case), the proposed framework achieved a mean absolute error of approximately 8–9 mmHg for SBP and 6–7 mmHg for DBP, reflecting the intrinsic difficulty of cross-subject generalization without prior calibration.

### 3.5. Monitoring Scenario with Prior Calibration

To evaluate the capability of the proposed Smart Sock system for longitudinal blood pressure monitoring after calibration, a second evaluation protocol was implemented using stratified window-level cross-validation. In this setting, signal windows from the same participants are allowed to appear in both training and testing folds, reflecting a realistic use case in which one-time calibration is performed for each user and subsequently tracks BP continuously over time.

To prevent bias toward dominant pressure ranges, stratification was performed using derived blood pressure categories corresponding to established clinical thresholds (normal, elevated, stage 1, and stage 2 hypertension). This ensured a balanced representation of physiological states across folds and maintained consistent exposure of both normotensive and hypertensive conditions during model training and evaluation. Consequently, performance metrics obtained under this protocol reflect the system ability to track intra-subject blood pressure variations across a wide range of clinically relevant values rather than overfitting to a narrow operating range.

**Figure 10:**
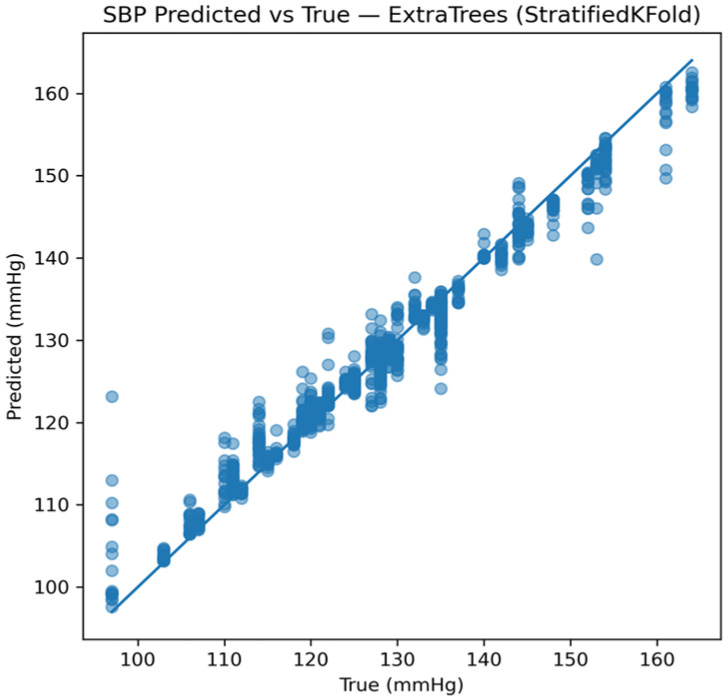
Window-level predicted versus reference SBP using the Extra Trees model under the calibrated monitoring scenario.

Model performance was first evaluated at the window level. Figure 10 presents representative predicted-versus-true SBP values for the best-performing model under this monitoring protocol. The tight clustering of predictions around the identity line indicates strong agreement between estimated and reference values once subject-specific calibration is available.

**Figure 11:**
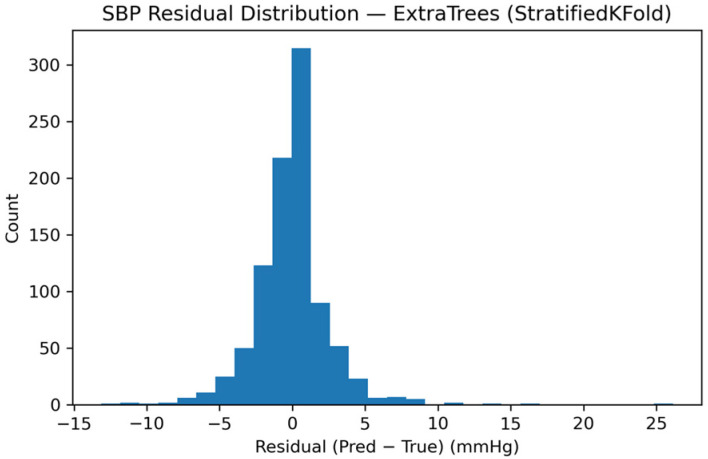
Distribution of SBP residuals (prediction minus reference) for the calibrated monitoring scenario.

Residual behavior was further examined to assess error distribution and bias. Figure 11 presents the residual histogram for SBP, demonstrating a narrow, approximately symmetric error distribution centered near zero, consistent with high-precision tracking.

**Figure 12:**
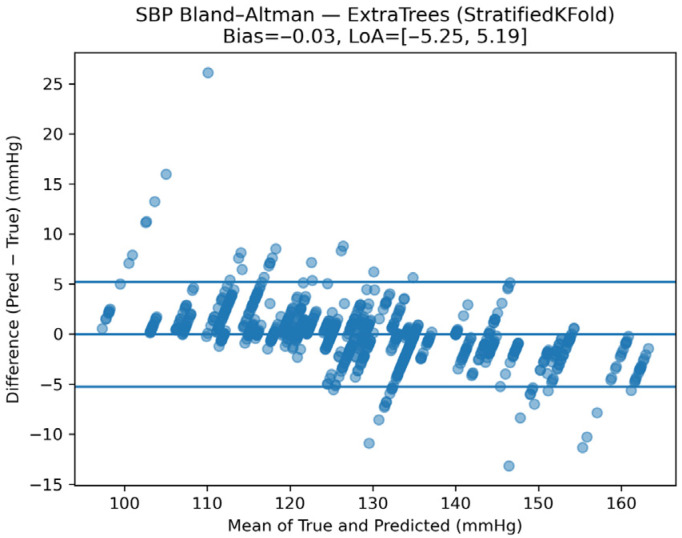
Bland–Altman analysis of SBP estimates under the calibrated monitoring scenario.

Agreement analysis was conducted using Bland–Altman plots. Figure 12 illustrates the Bland–Altman analysis for SBP, showing minimal systematic bias and narrow limits of agreement, indicating stable and clinically acceptable tracking performance.

**Figure 13:**
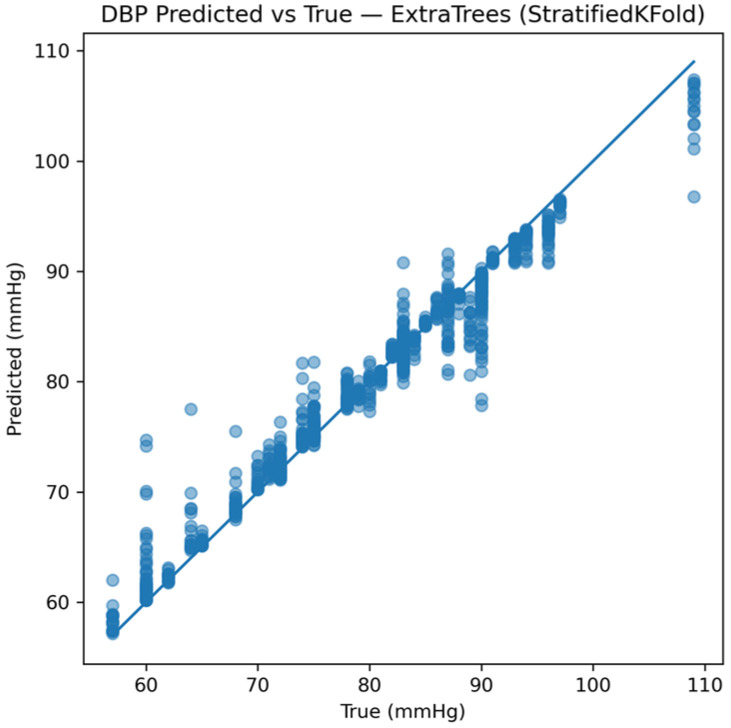
Comparison between predicted and true diastolic blood pressure (DBP) values at the single time-window level, highlighting the prediction accuracy of the system.

**Figure 14:**
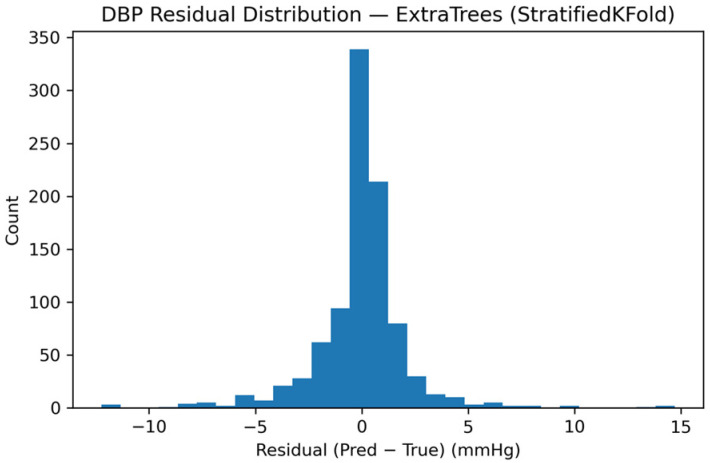
Residual distribution of the DBP predictions, showing the difference between model-estimated and reference values to assess the precision of the Smart Sock system.

**Figure 15:**
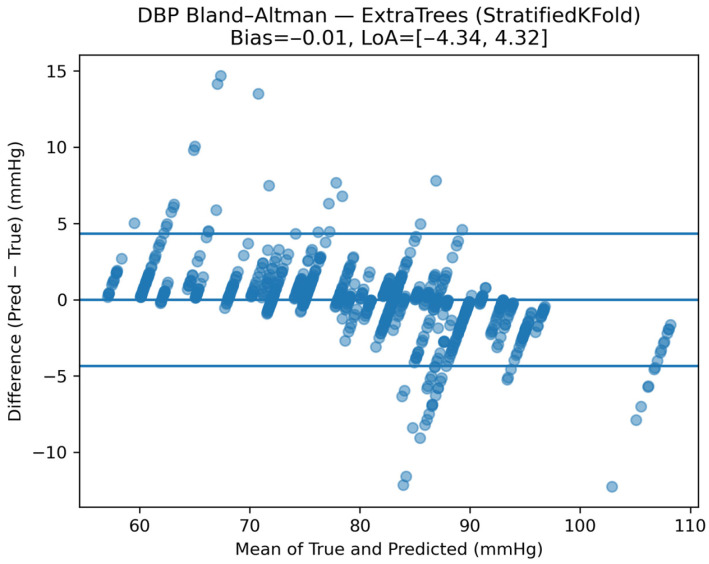
Window-level DBP prediction accuracy and agreement under the calibrated monitoring scenario, assessed via Bland–Altman analysis between the Smart Sock system and reference cuff measurements. Across models, the calibrated monitoring scenario achieved substantially improved accuracy compared to subject-independent evaluation. Mean absolute error values ranged from approximately 1.3–1.7 mmHg for both SBP and DBP, with coefficients of determination (R^2^) consistently exceeding 0.90, indicating a highly precise subject-specific BP tracking after calibration.

Equivalent trends were observed for DBP. Figure 13, Figure 14 and Figure 15 show predicted-versus-true values, residual distributions, and Bland–Altman agreement plots for DBP, confirming consistent performance across both pressure components.

### 3.6. Summary of Results

The experimental results demonstrate that the proposed Smart Sock framework supports two clinically distinct use cases for cuffless BP monitoring. In the diagnostic scenario, subject-independent evaluation produces moderate errors, consistent with the known challenges of cross-subject generalization using peripheral PPG signals. In contrast, the calibrated monitoring scenario enables highly accurate BP tracking once individual vascular characteristics are learned.

Key contributors to this performance include the use of peak-independent features derived from PPG, VPG, and APG signals, as well as the explicit incorporation of temporal lag features that model short-term hemodynamic memory over 5–15 s intervals. This design allows the learning models to capture baseline shifts, vascular tone dynamics, and activity-related variability without relying on fiducial pulse timing or pulse transit time estimation.

Finally, these results highlight the importance of evaluation protocol selection when reporting wearable BP performance and suggest that foot-based PPG, combined with temporal context modeling, provides a robust and physiologically meaningful foundation for continuous, real-world cardiovascular monitoring.

## 4. Discussion

### 4.1. Physiological Interpretation of Foot-Based PPG Features

The results of this study confirm that foot-based PPG provides physiologically meaningful information for cuffless blood pressure estimation when appropriate feature representations are used. Compared more with proximal sensing sites such as the finger or wrist, the lower limb represents a distal vascular territory, where pulse waveforms are more strongly influenced by arterial stiffness, wave reflections, and peripheral resistance. These effects are particularly evident in the APG of the PPG signal, which emphasizes inflection points and reflects wave components that are closely associated with vascular aging and arterial compliance.

By combining features extracted from the PPG signal and its VPG and APG, the proposed framework captures complementary aspects of blood volume, flow velocity, and acceleration dynamics. This multi-order representation is particularly advantageous for distal measurements, where reflected waves dominate morphology and simple amplitude-based descriptors are insufficient. The main contribution of APG-based skewness and dispersion features observed in the permutation importance analysis further supports the physiological relevance of acceleration-domain information for BP estimation at the foot.

### 4.2. Role of Temporal Lag Modeling and Hemodynamic Memory

A central contribution of this work is the explicit modeling of short-term hemodynamic memory through the construction of a temporal lag matrix. Rather than treating each analysis window as an independent observation, selected features were augmented with delayed versions spanning 5–15 s. This design enables the learning algorithms to capture gradual baseline shifts and vascular tone trends that occur over multiple cardiac cycles.

From a physiological point of view, this temporal context is consistent with known regulatory mechanisms such as the baroreflex, which modulates blood pressure over several seconds rather than instantaneously. By embedding this temporal structure directly into the feature space, the proposed approach can be considered physics-compliant, as it respects the inherent dynamics of cardiovascular control rather than relying solely on static waveform snapshots. Importantly, this strategy avoids explicit pulse peak detection and timing fiducials, which are often unreliable in ambulatory conditions, while still preserving temporal information critical for accurate BP estimation.

### 4.3. Diagnosis Versus Monitoring Scenarios: Interpretation of Performance

The distinction between subject-independent diagnosis and subject-specific monitoring is essential for interpreting cuffless blood pressure performance. In the diagnosis scenario, GroupKFold cross-validation enforced strict subject separation between training and testing data, simulating first-time use of the Smart Sock without prior calibration. Under this protocol, subject-level aggregation resulted in mean absolute errors of approximately 8–9 mmHg for SBP and 6–7 mmHg for DBP. These values reflect the substantial inter-individual variability in vascular properties and are consistent with the difficulty of absolute BP estimation across unseen subjects reported in prior studies.

In contrast, the monitoring scenario represents longitudinal tracking after a single calibration event. Stratified window-level cross-validation allowed data from the same individual to appear in both training and testing folds, while preserving balanced BP category distributions. Under this calibrated setting, the Smart Sock achieved substantially improved accuracy, with MAE values in the range of 1.3–1.7 mmHg and coefficients of determination ($R^{2}$) exceeding 0.90 for both SBP and DBP. These results demonstrate that once individual vascular characteristics are learned, the system can track BP changes with high precision, as evidenced by the strong agreement between predicted and reference values.

This dual evaluation strategy provides an important interpretation of the results: while subject-independent BP estimation remains challenging for any single-site PPG system, the proposed Smart Sock performs better in its primary intended role as a personalized, continuous monitoring device.

The very low error values observed in the calibrated monitoring scenario should be interpreted in the context of short-duration, resting recordings, where true blood pressure variability is limited. In this setting, the proposed framework effectively performs noise-robust regression anchored to a subject-specific reference measurement, rather than estimating large physiological BP excursions.

### 4.4. Comparison with State-of-the-Art Wearable BP Systems

Previous work on cuffless blood pressure estimation has largely relied on PPG and PTT as core physiological metrics [3,8,10]. Smartphone-based approaches [11] and single-site PPG systems show promise but often require active user interaction or suffer from sensitivity to motion artifacts and inconsistent sensor–skin contact during daily activities [4,5]. ECG–PPG systems can improve timing accuracy by introducing a proximal cardiac reference, yet they increase hardware complexity and reduce long-term wearability [7].

The lower limb has been recognized as a robust anatomical site for vascular sensing [13], offering improved mechanical stability during daily movement. The proposed Smart Sock capitalizes on this advantage by combining foot-based PPG with peak-independent feature extraction and temporal modeling. In contrast with ECG–PPG systems that depend on precise fiducial alignment, the present framework leverages waveform morphology and short-term temporal dynamics, enhancing robustness in real-world conditions.

Table 1 highlights key distinctions between the proposed Smart Sock framework and existing cuffless BP monitoring approaches. Unlike ECG–PPG systems, which rely on explicit fiducial timing and synchronized multi-sensor measurements, the present work adopts a peak-independent feature representation derived from PPG and its derivatives. This design eliminates sensitivity to peak detection errors while preserving physiological interpretability through volume, velocity, and acceleration dynamics.

Compared with conventional single-site PPG systems, the Smart Sock benefits from the mechanical stability of the lower limb and explicitly models short-term hemodynamic memory via a temporal lag matrix. This enables robust performance during daily activities and supports personalized BP monitoring with minimal hardware complexity.

### 4.5. Limitations and Future Directions

Several limitations deserve consideration. First, the cohort consisted primarily of healthy adults; therefore, future validation in hypertensive, elderly, and clinical populations is required to assess generalizability across broader cardiovascular phenotypes.

Second, PPG data were acquired exclusively under resting conditions. While this controlled protocol was necessary to ensure high signal quality and to isolate vascular morphology effects from motion artifacts, it does not capture the challenges associated with ambulatory or physically active scenarios. Evaluation under dynamic conditions remains an important direction for future investigation.

Third, although the proposed monitoring framework demonstrates excellent performance following calibration, subject-independent accuracy remains limited by inter-individual vascular variability.

Finally, the present work focused on single-site PPG acquisition; integrating additional contextual signals, such as inertial or temperature data, may further enhance robustness under free-living conditions.

Future work may investigate adaptive calibration strategies, long-term drift compensation, and deployment in uncontrolled environments. Nevertheless, the present findings establish a strong foundation for foot-based, physics-compliant cuffless BP monitoring using peak-independent features and temporal lag modeling.

## 5. Conclusions

This study introduced a novel foot-based Smart Sock platform for cuffless BP monitoring based on a peak-independent feature extraction framework. By shifting the sensing location to the anatomically stable plantar region of the foot and avoiding reliance on fragile fiducial timing features, the proposed system is inherently better aligned with the practical constraints of wearable and ambulatory monitoring.

The result demonstrates that statistical, energy-based, and information-theoretic descriptors extracted from the PPG signal and its VPG and APG derivatives provide a deep and physiologically meaningful representation of lower-limb hemodynamics. This feature engineering strategy eliminates dependence on error-prone peak detection while preserving sensitivity to vascular stiffness and wave reflection phenomena that are particularly prominent in distal arterial sites. Furthermore, the explicit modeling of short-term hemodynamic memory through the Temporal Lag Matrix proved to be critical for stable blood pressure estimation. By incorporating up to 15 s of feature history, the learning algorithms were able to account for gradual baseline shifts and vascular adaptations that cannot be captured by isolated signal windows, resulting in more consistent predictions over time.

Two clinically distinct evaluation paradigms were investigated. In the diagnosis (uncalibrated) scenario, subject-independent evaluation yielded a mean absolute error of 8.60 mmHg for SBP, reflecting the inherent challenge associated with inter-subject variability in single-site PPG-based systems. In contrast, the monitoring (calibrated) scenario demonstrated substantially higher precision, achieving MAE values in the range of 1.3–1.7 mmHg. These findings confirm the suitability of the proposed system for longitudinal blood pressure tracking following a single reference measurement.

From a system-level perspective, the Smart Sock framework reduces hardware complexity compared to ECG–PPG solutions while offering improved anatomical stability and wearability relative to conventional wrist-based PPG devices. The combination of foot-based sensing, peak-independent features, and temporal modeling provides a balanced trade-off between robustness, physiological interpretability, and practical deployability.

Overall, this work establishes a strong foundation for unobtrusive and physiology-personalized blood pressure monitoring, particularly in longitudinal scenarios following a single calibration measurement, using smart textile platforms. Future work will focus on validation under free-living conditions, multimodal context integration and adaptive calibration strategies to further mitigate subject-to-subject variability.

## Figures and Tables

**Table 1:** Comparison of the proposed Smart Sock with state-of-the-art cuffless BP monitoring approaches.

Metric	Standard Single-Site PPG [4,5,14]	ECG-PPG Systems [3,7,10,13]	This Work (Foot PPG)
Primary sensing modality	Single-Site PPG (finger/wrist)	ECG + proximal/distal PPG	Single-site foot PPG (Smart Sock)
Dependence on fiducial timing	Often uses peak/foot detection	Explicit timing (R-peak + PPG) à PTT	None (peak-independent features)
Motion robustness	Limited by hand/wrist motion	Moderate (ECG sensitive to motion)	High (lower-limb placement; wearable textile)
Anatomical stability	Peripheral; contact variability	Chest + limb synchronization required	Lower limb; mechanically stable location
Temporal modeling	Often single-window only	Beat-to-beat timing features	Lag matrix (5 s, 10 s, 15 s history)
Hardware complexity	Low	High (multi-sensor + electrodes)	Low (single-site PPG; sock-integrated)
Real-world wearable fit	Watch/handheld (user dependent)	Less comfortable for long-term wear	Comfortable textile wearable sock
Calibration requirement	Usually required	Usually required	Diagnosis: none; monitoring: single cuff calibration improves accuracy
Primary clinical role	Screening/wellness	Clinical-grade monitoring (but bulky)	Everyday monitoring + longitudinal tracking

## Data Availability

Due to participant privacy considerations and the ongoing commercialization of the Smart Sock platform, the raw physiological dataset is not publicly available. However, representative sample data, feature definitions, and signal processing scripts supporting the findings of this study are available from the corresponding author upon reasonable academic request to ensure methodological transparency and reproducibility.
